# *Salmonella* Trafficking is Defined by Continuous Dynamic Interactions with the Endolysosomal System

**DOI:** 10.1111/j.1600-0854.2006.00529.x

**Published:** 2007-01-15

**Authors:** Dan Drecktrah, Leigh A Knodler, Dale Howe, Olivia Steele-Mortimer

**Affiliations:** Laboratory of Intracellular Parasites, Rocky Mountain Laboratories NIAID, NIH, Hamilton, MT 59840, USA

**Keywords:** acidification, confocal, *Coxiella*, endosome, lysosome, microtubules, phagosome, SCV, type III secretion, V-ATPase

## Abstract

Following invasion of non-phagocytic host cells, *Salmonella enterica* survives and replicates within a phagosome-like compartment known as the *Salmonella*-containing vacuole (SCV). It is now well established that SCV biogenesis, like phagosome biogenesis, involves sequential interactions with the endocytic pathway. However, *Salmonella* is believed to limit these interactions and, in particular, to avoid fusion of terminal lysosomes with the SCV. In this study, we reassessed this process using a high-resolution live-cell imaging approach and found an unanticipated level of interaction between the SCV and the endocytic pathway. Direct interactions, in which late endosomal/lysosomal content was transferred to SCVs, were detected within 30 min of invasion and continued for several hours. Mechanistically, these interactions were very similar to phagosome–lysosome fusion because they were accompanied by rapid acidification of the SCV, could be blocked by chemical perturbation of microtubules or vacuolar acidification and involved the small GTPase Rab7. In comparison with vacuoles containing internalized *Escherichia coli* or heat-killed *Salmonella*, SCVs did show some delay of fusion and acidification, although, this appeared to be independent of either type III secretion system. These results provide compelling evidence that inhibition of SCV–lysosome fusion is not the major determinant in establishment of the *Salmonella* replicative niche in epithelial cells.

The facultative intracellular pathogen *Salmonella enterica* serovar Typhimurium is a frequent cause of food-borne gastroenteritis in humans. Invasion of non-phagocytic cells, such as intestinal epithelial cells, is an important virulence determinant that has been extensively studied *in vitro*. These studies have revealed a complex host–pathogen relationship that is mediated largely by two type III secretion systems (T3SS1 and T3SS2), which *Salmonella* uses to translocate bacterial effector proteins directly into the host cell [reviewed by Hueck (1), Hensel (2) and Marcus et al.(3)]. Several T3SS1 effectors cooperatively mediate invasion by inducing massive localized active rearrangements and ruffling of the plasma membrane. Once within the host cell the bacteria reside and replicate within a modified phagosome or *Salmonella*-containing vacuole (SCV). Both T3SS1 and T3SS2 contribute towards biogenesis of the SCV and establishment of the intracellular niche, however, despite intense investigation over the last decade, the mechanisms involved remain unclear [reviewed by Knodler and Steele-Mortimer (4), Patel and Galan (5) and Brumell and Grinstein (6)].

Comparison of SCV biogenesis with that of phagosomes (6), which has been described in detail, reveals many similarities and a few salient differences (Figure 1). Both vacuoles initially acquire early endosomal (EE) markers, including Rab5 (7), the transferrin receptor (TfnR) and EEA1 (early endosome associated antigen) (8), which are rapidly replaced by late endosome/lysosome (LE/Lys) markers, particularly Rab7, lysosomal membrane glycoproteins (lgps) such as LAMP1 (lysosomal associated membrane protein 1), LAMP2 and LAMP3, and the vacuolar proton pump (V-ATPase) (8–10). Biogenesis of both also involves the Rab11-regulated endocytic-recycling pathway (11,12). However, in some respects, the SCV apparently deviates from the default phagosome maturation pathway. For example, certain LE/Lys markers do not accumulate in the SCV or accumulate with different kinetics compared to phagosomes. In particular, several studies have found that lysosomal markers delivered by the calcium-independent mannose-6-phosphate receptors (CI-M6PR) as well as fluid-phase markers do not accumulate in SCVs in epithelial cells (9,13) or RAW 264.7 macrophage-like cells (14,15).

**Figure 1:**
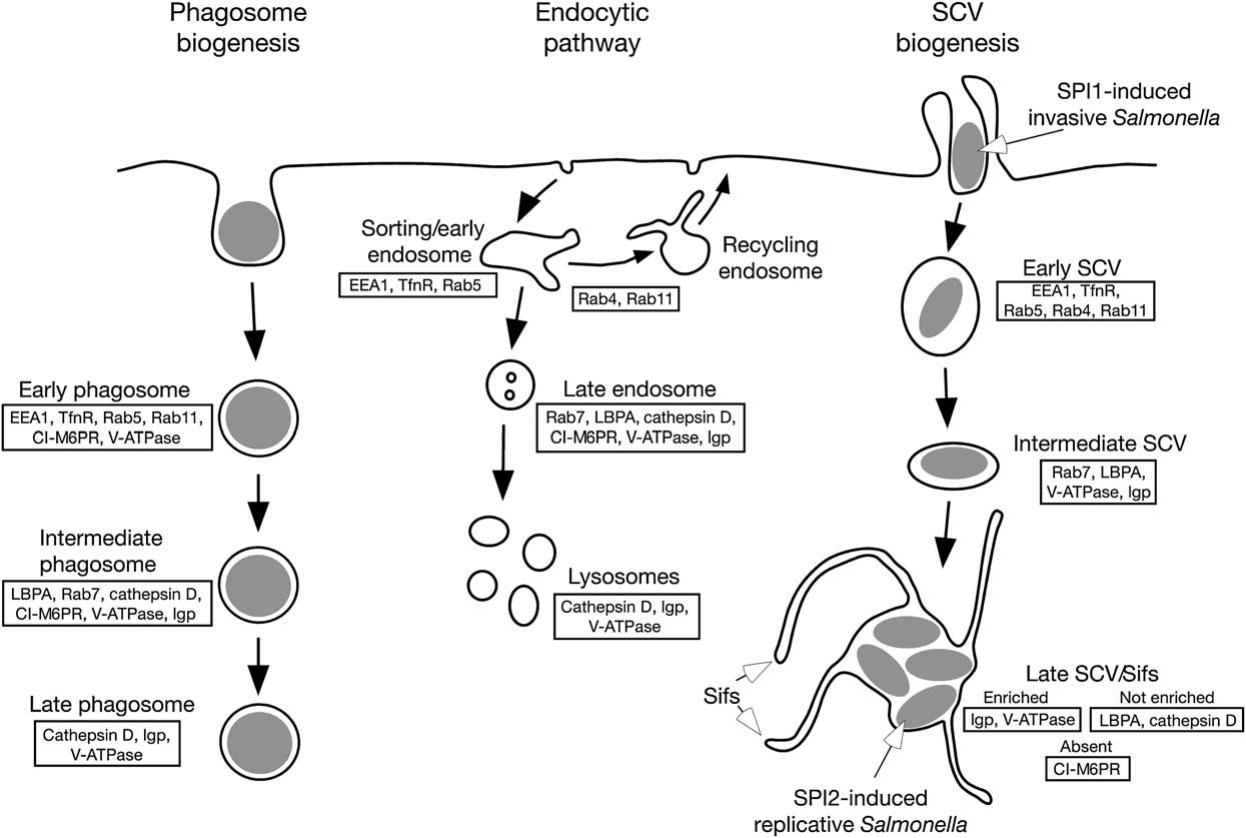
Markers of the endocytic pathway and their distribution during SCV maturation According to the prevailing model, the SCV transiently acquires markers of early endosomes and late endosomes during biogenesis, but delays delivery of the lysosomal hydrolase cathepsin D and lysobisphosphatidic acid (LBPA) and prevents the accumulation of CI-M6PRs compared to maturation of phagosomes. The SCV is also enriched in certain components of the endolysosomal pathway, particularly the V-ATPase and lgps. Markers of the late SCV also localize to membrane tubules termed *Salmonella*-induced filaments (Sifs).

Despite widespread acceptance for a model in which *Salmonella* block lysosome–SCV fusion, there are some indications that this may not be entirely accurate. One study in particular found that SCVs rapidly fuse with lysosomes in cultured macrophages, and concluded that *Salmonella* survive within a phagolysosome (16). Another group using RAW 264.7 cells also concluded that SCVs fuse with lysosomes although the extent of fusion was increased in the presence of the host-protective protein SLC11A1 (Nramp1) (15). This suggests that increasing lysosomal fusion with the SCV might be one way by which SLC11A1 prevents the replication of *Salmonella* in macrophages. Comparisons are complicated not only by the presence or absence of antibacterial host factors such as SLC11A1 but also by the fact that, in phagocytic cells at least, the mechanism of entry can also affect SCV biogenesis (17). Other factors, from infection protocols to cell lines, may also account for variation in results from different laboratories.

In this study we have re-examined the interactions of the SCV with the endocytic pathway in epithelial cells. Unlike macrophages, which can internalize *Salmonella* via several distinct mechanisms, non-phagocytic epithelial cells only internalize invasive, that is, T3SS1-induced, *Salmonella*(18). Intestinal epithelial cells, which do not express SLC11A1, are a crucial intracellular niche for *Salmonella*(19) and the question of whether the SCV truly segregates from the endocytic/lysosomal system in such cell types is important. Here we show that the SCV is freely accessible to fluorescent fluid-phase markers from both preloaded LE/Lys and incoming endocytic traffic. To validate this finding, we confirmed that SCV biogenesis and the endocytic pathway have several functional similarities; rapid luminal acidification mediated by the V-ATPase as well as involvement of microtubules and the small guanosine triphosphatase (GTPase), Rab7. In conclusion, these findings suggest that, contrary to the current hypothesis, SCV biogenesis involves extensive dynamic interactions with the endocytic pathway including LE/Lys.

## Results

### The SCV in epithelial cells is accessible to fluid-phase markers from both lysosomes and incoming endocytic vesicles

The current model for SCV biogenesis holds that SCVs rapidly segregate from the endocytic pathway and do not fuse with lysosomes, a hypothesis that was developed after several early studies found that the SCV in epithelial cells was inaccessible to fluid-phase markers from preloaded lysosomes (9) or incoming endocytic vesicles (13,20). As the question of whether or not SCVs interact with LE/Lys is crucial to our understanding of *Salmonella* pathogenesis, we have readdressed this question using high-resolution live-cell confocal microscopy to assess transfer of content from the endocytic pathway to the SCV. Crucially, this technique avoids the considerable loss of fluid-phase marker (>90%) that occurs during fixation and processing steps (Figure S1). In addition, because epithelial cells, in comparison with professional phagocytes, are extremely inefficient at internalizing fluid-phase probes, we also used a longer internalization protocol in which HeLa cells were incubated with fluorescent dextrans overnight (o/n) followed by a 3 h chase instead of the 3–4 h internalization and o/n chase used previously (9). To verify that this modification did not change the proportion of endosomal versus lysosomal labeling, control experiments were carried out in which two fluid-phase markers, Alexa 488-conjugated dextran (dextran-488) and Alexa 568-conjugated dextran (dextran-568), were internalized and their distributions assessed in live cells (Figure S1). Full co-localization (100% of dextran-568 overlapping with dextran-488) was observed, as expected, when the dextrans were internalized together using the o/n chase protocol, and was only slightly reduced (93 ± 3%) when dextran-568 was internalized o/n after a 3 h pulse of dextran-488. In contrast, when dextran-568 was internalized for 20 min immediately before live-cell imaging there was a significant reduction in the amount of co-localization (50 ± 14%). Similar results were obtained from live-cell experiments using cells expressing the LE/Lys membrane marker LAMP1–monomeric green fluorescent protein (mGFP) instead of internalized dextran-488. Specifically, there was no significant difference in co-localization of dextran-568 with LAMP1–GFP regardless of whether it was internalized for 3 h and chased o/n (97 ± 4%) or internalized o/n with a 3 h chase (93 ± 3%). Altogether these experiments showed that an o/n internalization followed by a 3 h chase can be used to label LE/Lys.

**Figure 2:**
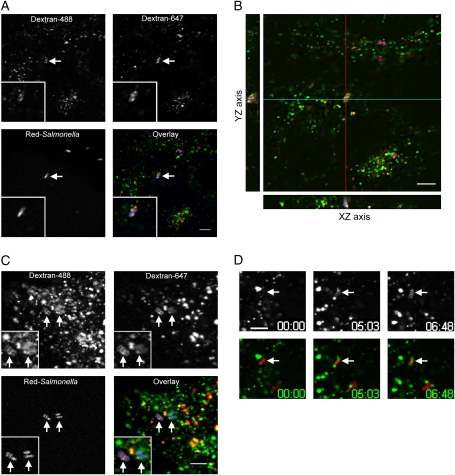
SCVs acquire content from endosomes and lysosomes To load lysosomes HeLa (A, B and D), or C2BBe1 (C), cells were preincubated with dextran-647 (red) o/n, then chased in dextran-free media for 3 h before infection with Red-*Salmonella* (blue). After infection, cells were incubated with dextran-488 (green) to load endosomes and live-cell imaging was initiated at 3 h p.i. A) A single confocal plane showing co-localization of dextran-647 and dextran-488 in a single SCV (arrow). B) A projection of 36 focal planes with YZ (red line) and XZ (blue line) side views to show co-localization of dextrans. C) A single confocal plane from the center of Red-*Salmonella*-infected C2BBe1 epithelial cells, showing co-localization of dextran-647 and dextran-488 in individual SCVs (arrows). D) Selected frames from a time-lapse series showing delivery of dextran-488 (top) from preloaded lysosomes to an SCV (arrows). Image acquisition started 15 min p.i. (00:00). Scale bars = 5 μm.

Having established conditions for optimal labeling and imaging of LE/Lys compartments in HeLa cells, we next used this system to reassess the accessibility of SCVs to LE/Lys content. For live-cell imaging of *Salmonella*, we used bacteria constitutively expressing monomeric DsRed (Red-*Salmonella*) (17) because these bacteria could be imaged with LAMP1–mGFP or dextran-488 as well as Alexa 647–conjugated dextran (dextran-647) on our system. LE/Lys were first labeled by o/n internalization of dextran-647 followed by a 3 h chase, then the cells were infected with T3SS1-induced Red-*Salmonella* at a multiplicity of infection (MOI) ∼ 20 for 10 min. This infection protocol results in a highly synchronous infection with 0–10 bacteria internalized per cell (not shown). Two hours post-infection (p.i.), dextran-488 was added to the medium, as a marker of incoming endocytic content, and live-cell imaging was initiated 1 h later. Figure 2 shows, contrary to previous results (9), accumulation of both LE/Lys and incoming markers in the SCV. This accumulation of dextrans in the SCV is better visualized in the YZ and XZ axis of a three-dimensional (3D) reconstruction (Figure 2B, see also video *Supplementary Material*, Video S1). Quantification of these experiments revealed that 56 ± 2% of SCVs contained markers from both preloaded LE/Lys and incoming endocytic traffic at 6 h p.i. and that 100% of SCVs containing the incoming fluid-phase marker were also positive for the LE/Lys marker (Figure 2 and data not shown). Time-lapse imaging revealed that delivery of LE/Lys content to SCVs is a dynamic process involving multiple physical interactions leading to the gradual accumulation of marker with time (Figure 2D arrow and see also video *Supplementary Material*, Video S2). To ensure that this was not a cell type–specific phenomenon, we examined the accessibility of the SCV in a colonic epithelial cell line (C2BBe1). As in HeLa cells, both LE/Lys and incoming endocytic markers were found to accumulate in SCVs (Figure 2C).

**Figure 3:**
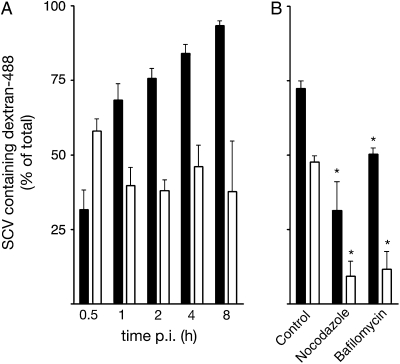
SCV acquisition of fluid-phase content from endosomes and lysosomes is dependent on V-ATPase and microtubules To load lysosomes, HeLa cells were preincubated with dextran-488 o/n, then chased in dextran-free media for 3 h before infection with Red-*Salmonella*. To follow endocytic access to the SCV dextran-488 was added various times after infection. Cells were then observed by confocal microscopy. Images of dextran-488 and Red-*Salmonella* were overlaid and the number of SCVs containing dextran-488 were counted. A) Time course of SCV interaction with terminal lysosomes (filled bars) and incoming endocytic traffic (open bars). B) To assess the effect of microtubule depolymerization or V-ATPase inhibition on marker acquisition 20 μM nocodazole or 0.5 μM bafilomycin was added, respectively, 1 h before infection and maintained throughout the experiment. Live-cell imaging was initiated at 2 h p.i. and SCVs containing dextran-488 were counted. Values are the mean ± SD of three independent experiments (*n* ≥ 60 in each experiment). Asterisk indicates significant difference from control-treated cells (p < 0.05, anova, Tukey’s *post hoc* test).

To quantify SCV accessibility to the endocytic pathway, we counted SCVs containing detectable amounts of dextran-488 in live cells over an infection time–course. By 30-min p.i., 30% of SCVs contained marker from preloaded LE/Lys and this increased to 90% by 8 h p.i. (Figure 3A, filled bars). In comparison, 40–60% of SCVs contained detectable amounts of dextran-488 from incoming endocytic traffic at all time points during the SCV maturation process up to 8 h p.i. (Figure 3A, open bars). Overall, these experiments reveal an unanticipated level of interaction between SCVs and the endocytic pathway.

### SCV acquisition of endosome/lysosome content is dependent on microtubules and vacuolar acidification

To investigate the mechanism of delivery of endosome content to SCVs, we next took advantage of two well-described pharmacological inhibitors of the endocytic pathway, bafilomycin A1 and nocodazole. Bafilomycin A1, an inhibitor of the V-ATPase, raises the pH of endosomes to neutrality in HeLa cells and selectively inhibits the transport of markers destined for lysosomal delivery in the early endosome (21,22). In contrast, nocodazole is a microtubule-disrupting drug that blocks recycling markers, such as transferrin, in the recycling compartment and lysosomally targeted markers in endocytic carrier vesicles (21). We hypothesized that both inhibitors should block trafficking of incoming endocytosed markers to preformed SCVs because the markers will be trapped in either early endosomes or endosomal carrier vesicles. Accordingly, we found that dextran internalized in the presence of either inhibitors was delivered inefficiently to preformed SCVs (Figure 3B, open bars). Both inhibitors also decreased delivery of dextrans from preloaded lysosomes to the SCV, though bafilomycin A1 was less effective at inhibiting this step (Figure 3B, closed bars). Together these data provide strong evidence that a functionally active endocytic pathway is required for efficient delivery of LE/Lys and endosomal content to SCVs.

### SCV acquisition of endosome/lysosome content is Rab7 dependent

**Figure 4:**
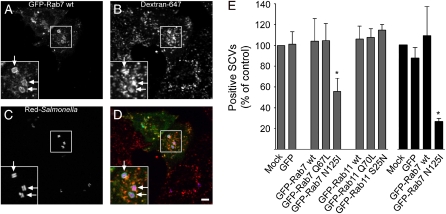
SCV acquisition of fluid-phase markers is Rab7 dependent HeLa cells expressing GFP alone or GFP-Rab protein fusions (green), were infected with Red-*Salmonella* (blue). Dextran-647 (red) was added 2 h p.i. to load the endocytic pathway. Live-cell imaging was initiated 1 h after addition of dextran-647. A single confocal plane is shown (D, overlay) to illustrate the presence of dextran-647 (B) in SCVs containing Red-*Salmonella* (C) that are outlined by GFP-Rab7 wt (A). Scale bar = 5 μm. E) Quantification of dextran-647 and V-ATPase positive SCVs. SCVs containing dextran-647 (gray bars) were scored in GFP-expressing cells and expressed as percentage of dextran–647 positive SCVs in mock-transfected cells (set to 100%). For quantification of V-ATPase accumulation on the SCV, transfected cells (black bars) were infected with Red-*Salmonella* then fixed and processed for immunofluorescence 1 h p.i. V-ATPase was detected using mouse monoclonal antibodies followed by Cy5-conjugated donkey α-mouse antibodies. SCVs staining positive for V-ATPase were counted in GFP-expressing cells and expressed as percentage of V-ATPase positive SCVs in mock-transfected cells (set to 100%). Values are the mean ± S.D. of three independent experiments (*n* ≥ 50 in each experiment). Asterisk indicates significant difference from all other conditions (p < 0.05, anova, Tukey’s *post hoc* test).

Having shown that the SCV is accessible to the endocytic pathway, we next examined the role of the small GTPases, Rab7 and Rab11 both of which participate in trafficking, along the endocytic pathway (23) and have previously been implicated in SCV biogenesis (10,11). GFP-Rab7 and GFP-Rab11 were transiently expressed in HeLa cells, which were then infected with Red-*Salmonella*. Two hours p.i., dextran-647 was internalized for 1 h, and the percentage of SCVs containing marker was then assessed by live-cell confocal microscopy (Figure 4A–D, arrows). Figure 4E shows that, compared to mock or GFP-transfected cells, expression of either Rab7 wild-type or the constitutively active mutant Rab7 Q67L had no effect on the number of SCVs containing detectable dextran-647, whereas the dominant negative mutant, Rab7 N125I, caused a decrease of 44 ± 12% in the number of SCVs containing dextran-647 compared to the control. Overexpression of Rab11, constitutively active Rab11 Q70L or dominant negative Rab11 S25N had no significant effect on the numbers of SCVs containing dextran-647 (Figure 4E). Thus Rab7, but not Rab11, is involved in the delivery of endosomal content to SCVs.

We next examined the effect of Rab7 on recruitment of V-ATPase to the SCV because SCVs acquire LAMP1 in a Rab7-dependent manner (10) and Rab7 protein has also been implicated in the acidification of phagosomes containing latex beads (24), a process mediated by V-ATPase. For these experiments, GFP-Rab7–transfected HeLa cells were infected with *Salmonella*, fixed at 1 h p.i. and then processed for indirect immunofluorescence microscopy using monoclonal antibodies against the V-ATPase to follow its intracellular localization. Overexpression of the Rab7–dominant negative mutant, Rab7 N125I, decreased V-ATPase delivery to the SCV (Figure 4E, black bars) confirming that both content and membrane proteins are delivered to the SCV in a Rab7-dependent manner.

### Increased lysosomal vesicle association with the SCV

**Figure 5:**
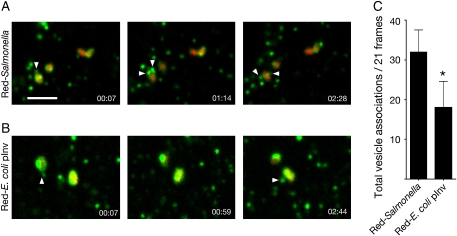
Increased lysosomal vesicle association with the SCV LE/Lys were loaded by incubating HeLa cells with dextran-488 o/n, followed by a chase in dextran-free media for 3 h before infection with Red-*Salmonella* or Red-*E. coli* pInv. Live-cell imaging was initiated at 45 min p.i. and time series of 3D image volumes were collected at a rate of 28.6 frames per minute for 3.5 min. Each image volume consisted of three optical planes, spaced 0.19 μm apart, for both the dextran-488 and the Red-bacteria. Projections of selected frames are shown for Red-*Salmonella* (A) and Red-*E. coli* pInv (B) infected cells showing the association of dextran–488 containing vesicles (green, arrowheads) with vacuoles containing the bacteria (red). See Video S3 for time-lapse movies of the SCV and Video S4 *E. coli* pInv vacuoles. The number of vesicles detected within 1 pixel (0.19 μm) of each bacterium was quantified (C). Values are expressed as the total number of vesicles associations per bacterium in 21 frames total (every fifth frame in 100 frame series) and represent the mean ± SD of three independent experiments (*n* ≥ 24). Asterisk indicates significant difference p < 0.05, Student’s *t*-test.

As the above results indicated that LE/Lys vesicles fuse with the SCV, but previous studies have suggested that this fusion event must be inhibited or delayed, we next investigated whether *Salmonella* modulates recruitment of LE/Lys to the SCV by live-cell imaging. Interaction of LE/Lys with SCVs was compared with that of phagosomes containing non-pathogenic *Escherichia coli* expressing Invasin from *Yersinia pseudotuberculosis*. Invasin binds the β_1_-integrin receptor and thus mediates internalization of non-invasive bacteria or inert particles when expressed on their surface (25–27). The LE/Lys interactions were directly visualized using internalized dextran-488 as a marker for lysosome content and fluorescent DsRedm-expressing bacteria. Dextran-labeled vesicles interacted with either SCVs or phagosomes containing Red-*E. coli* pInv via a dynamic process characterized by multiple physical interactions and fusion events, although one apparent difference was that dextran-containing lysosomes appeared in juxtaposition with SCVs more frequently than with phagosomes containing Red-*E. coli* pInv (Figure 5A,B arrowheads, see *Supplementary Material* videos S3 and S4). To quantify this phenomenon, time-lapse sequences were acquired and the number of distinct dextran-containing vesicles associated with SCVs/phagosomes (with no more than 1 pixel separating them) were counted in individual frames. As shown in Figure 5C, significantly more dextran-containing LE/Lys were found to be associated with SCVs compared with vacuoles containing *E. coli*. Together, these results suggest that, although SCVs fuse with LE/Lys, fusion may be delayed or restricted compared with phagosomes containing *E. coli*.

### SCV fuses with the parasitophorous vacuole of Coxiella burnetii

**Figure 6:**
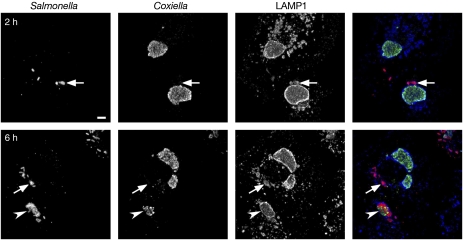
SCVs fuse with preformed *C. burnetii* parasitophorous vacuoles in co-infected cells HeLa cells were infected with *C. burnetii* for 48 h before infection with wt *Salmonella*. At 2 or 6 h p.i., cells were fixed and processed for immunofluorescence microscopy using α-*Salmonella* LPS (red), α-*Coxiella* (green) and α-LAMP1 (blue) antibodies. Projections of 12–15 confocal planes are shown. Arrows indicate *Salmonella* that are adjacent to, but not within, a *Coxiella* vacuole. An arrowhead indicates a vacuole that contains both *Salmonella* and *Coxiella*. Scale bar = 5 μm.

Intracellular pathogens that enter host cells via the endocytic pathway usually avoid lysosomal killing by interfering with the maturation process of the phagosome. However, some pathogens, such as the obligate intracellular pathogen, *C. burnetii*, inhabit vacuoles with many characteristics of phagolysosomes (28). Double-infection studies using *C. burnetii* together with other intracellular pathogens have shown that their localization within the same vacuole correlates with their ability to fuse with lysosomes. For example, the parasitophorous vacuoles of *Leishmania amazonensis*(29,30) and *Trypanosoma cruzi*(31) fuse with lysosomes and will also fuse with the *C. burnetii* vacuole. In contrast, vacuoles containing pathogens that segregate themselves from the endocytic pathway, such as *Chlamydia trachomatis*(28) and *Toxoplasma gondii*(32), do not fuse with the *C. burnetii* vacuole. To investigate which group *Salmonella* falls into we infected HeLa cells with *C. burnetii* and then 48 h later infected with wild-type (wt) *Salmonella*. Fusion of SCVs with preformed *C. burnetti* vacuoles was then monitored by immunofluorescence microscopy using LAMP1 as a marker for vacuole fusion because this lgp is highly enriched in the membrane of both vacuoles. In cells containing both pathogens, 15 ± 7% of *Salmonella* were localized in *C. burnetii* vacuoles at 2 h p.i., but the majority of *Salmonella* were in distinct SCVs as determined by the presence of an intact LAMP1-positive membrane (Figure 6, arrow in 2 h panel). By 6 h p.i., 39 ± 5% of *Salmonella* were colocalized with *C. burnetti* within a single LAMP1-positive membrane (Figure 6, arrowhead and see *Supplementary Material* Video S5). Thus *Salmonella*, like *L. amazonensis* and *T. cruzi*, do not prevent fusion with *C. burnetii* vacuoles.

### Acidification of the SCV is delayed in epithelial cells

**Figure 7:**
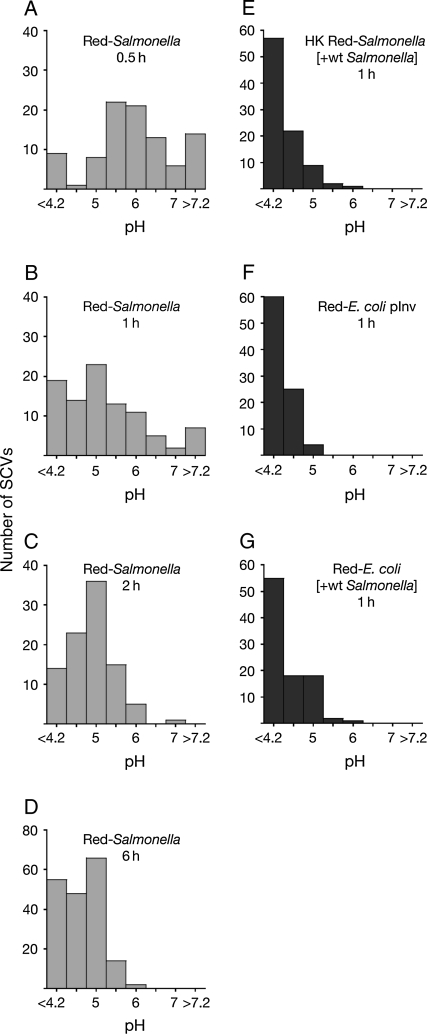
SCV acidification is delayed in epithelial cells The endolysomal system and vacuoles containing bacteria were loaded with dextrans by incubating HeLa cells o/n and throughout infection with a mixture of dextran-FITC and dextran-647. A–D) pH_SCV_ profiles from 0.5 to 6 h p.i. Live-cell imaging was initiated at the indicated time points after infection with Red-*Salmonella*. Sequential images (single optical planes) were obtained for the green (dextran-FITC), blue (dextran-647) and red (Red-*Salmonella*) channels. The gray scale images were combined into a single RGB image and SCVs containing Red-*Salmonella* and dextran-647 were selected. The fluorescence intensity ratio (488/647 nm) was acquired for each SCV and converted to pH_SCV_ using an *in situ* calibration curve obtained for each sample. E) The pH_VAC_ of HK Red-*Salmonella* taken up by co-internalization with wt *Salmonella*. F) The pH_VAC_ of Red-*E. coli* pInv. (G) The pH_VAC_ of Red-*E. coli* vacuoles taken up by co-internalization with wt *Salmonella*. The data are presented as histograms of pH_VAC_ or pH_SCV_ distributed in 0.5 unit bins. Each graph combines three independent experiments (*n* = 30 in each experiment).

Luminal acidification of endosomes, lysosomes and phagosomes is closely correlated with functionality. For example, low pH is required for full functionality of lysosomal hydrolases and the microbicidal function of phagosomes. Acidification is also believed to be a key stimulus for the intracellular induction of T3SS2 activity (33). The actual pH of the SCV lumen (pH_SCV_), however, has only been measured in macrophages where it decreases to <pH 5 within 1 h (15,34). Interestingly, the rate of acidification in macrophages does not seem to be significantly delayed by *Salmonella*(34), although our data, showing delayed delivery of lysosomal content in epithelial cells, suggest that this might not be the case in epithelial cells. To directly measure pH_SCV_ in HeLa cells, we used a ratiometric method based on the pH sensitivity of fluorescein [fluorescein isothiocyanate (FITC)] fluorescence (17). Cells were preloaded o/n with a mixture of dextran-FITC and pH-insensitive dextran-647 to label the entire endocytic pathway and then invasion was carried out in the presence of markers in order to optimize the concentration in SCVs. Using the spinning disk, confocal microscope images were acquired for the 568 nm (Red-bacteria), 488 nm (dextran-FITC) and 647 nm (dextran-647) channels and the 488:647 ratio obtained for individual SCVs as described previously (17). Initially, the pH_SCV_ was heterogeneous, ranging from >7.2 to <4.2, with a median of 5.9 at 0.5 h and 5.1 at 1 h p.i. (Figure 7). By 2 h p.i., the pH_SCV_ was more homogenous and acidic with the majority having a pH <5.2 and a median of 4.9, and remained this way for up to 6 h p.i. (median pH_SCV_ = 4.9). As shown previously, SCV acidification was abrogated in the presence of the V-ATPase inhibitor bafilomycin (34,35 and not shown).

We next compared pH_SCV_ to that of vacuoles containing Red-*E. coli* pInv. The Red-*E. coli* pInv vacuoles were more rapidly acidified, so that at 1 h p.i., the majority had a pH <4.8 (median pH <4.2) (compare Figure 7F and 7B). As Invasin-mediated internalization might itself account for the different rates of acidification, we also did co-infection experiments where heat-killed (HK) Red-*Salmonella* or live Red-*E. coli* were co-internalized with wt invasive *Salmonella*. In this way, the non-invasive bacteria are taken up into a vacuole by a process similar to SCV formation, although this is not a replicative environment (27). In contrast to SCVs containing live wt *Salmonella*, vacuoles containing co-internalized live or dead bacteria acidified rapidly and by 1 h p.i., the pH profiles were indistinguishable from vacuoles containing *E. coli* internalized via Invasin-mediated uptake (Figure 7E–G). Thus, SCV acidification is delayed in epithelial cells via a process that is not simply determined by the mechanism of uptake and presumably requires *Salmonella* factors that cannot act *in trans*.

### SCVs containing ΔSPI1 or ΔSPI2 mutants do not acidify more rapidly

**Figure 8:**
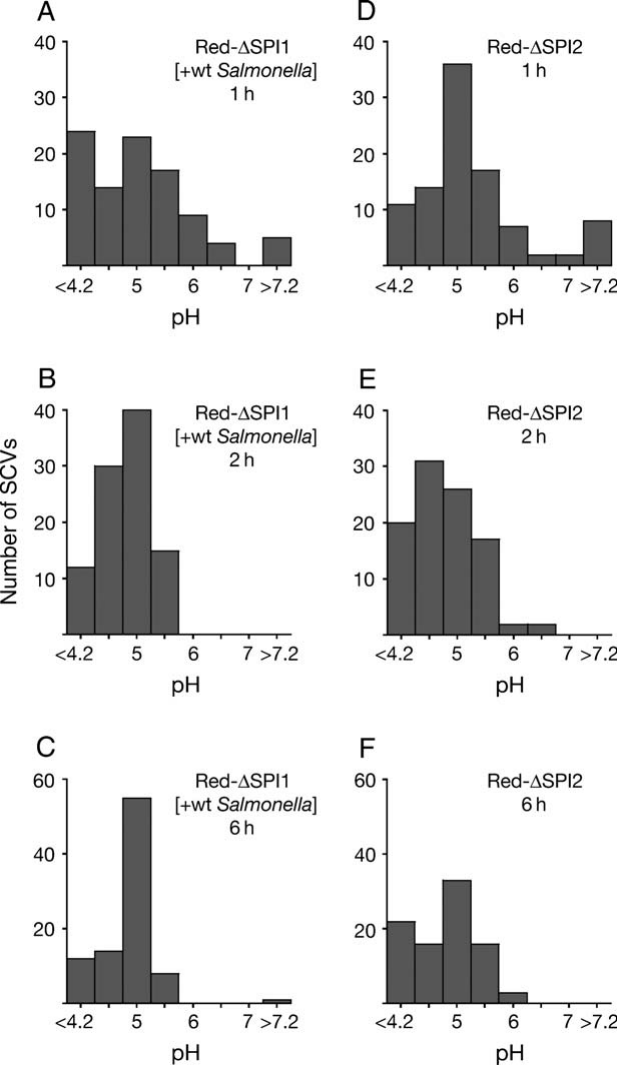
SCVs containing ΔSPI1 or ΔSPI2 mutants do not acidify more rapidly HeLa cells were loaded with dextran-FITC and dextran-647 as in Figure 7. A–C) Time course of the pH_SCV_ of ΔSPI1 Red-*Salmonella* taken up by co-internalization with wt *Salmonella*. D–F) Time course of the pH_SCV_ of ΔSPI2 Red-*Salmonella* internalized by SPI1-mediated invasion. The data are presented as histograms of pH_SCV_ distributed in 0.5 unit bins. Each graph combines three independent experiments (*n* = 30 in each experiment).

Intracellular survival of *Salmonella* and SCV biogenesis are dependent on effectors translocated by T3SS1 and T3SS2. To test if either of these systems is involved in the delay of SCV acidification, we used Red-*Salmonella* lacking the Salmonella Pathogenicity Islands, SPI1 or SPI2, that encode the structural components, regulators and some effector proteins of T3SS1 and T3SS2, respectively. As the ΔSPI1 mutant is non-invasive, it was internalized by co-infection with wt (non–DsRedm expressing) bacteria. Figure 8 shows that there was no significant difference in the pH_SCV_ profiles of ΔSPI1 or ΔSPI2 mutants and they were both indistinguishable from the pH_SCV_ profile of wt *Salmonella* (compare with Figure 7). These results suggest that neither of the T3SSs is involved in delaying SCV acidification.

## Discussion

Historically, the SCV has been described as a unique organelle whose biogenesis is actively directed by *Salmonella* in order to segregate from the endolysosomal system and thereby establish a replicative intracellular niche [reviewed by Brumell and Grinstein (6)]. Indeed, this model has become so well established that it is often presented as the paradigm for avoidance of lysosomal fusion even though other intracellular pathogens, such as mycobacteria, are clearly more proficient in this strategy of intracellular survival (36). However, while the prototypical model is accurate in that *Salmonella* can modify both the membrane composition of the SCV and its positioning within the host cell, the degree to which it is separated from the endocytic pathway remains ill-defined (15,37). Here, we have revisited the question of SCV–endolysosomal interactions using an established epithelial cell–infection model. We found that, contrary to popular perception, SCV biogenesis involves sustained dynamic interactions with the endocytic pathway, including LE/Lys and almost certainly terminal lysosomes. Specifically, the SCV obtains content from LE/Lys and remains accessible to incoming endocytic traffic. These interactions with the endolysosomal system involve Rab7, microtubules and the V-ATPase.

Against the backdrop of these findings, it is easy to wonder how such a sustained level of interaction with the endocytic pathway could have been overlooked for so long. In fact, several earlier studies did reveal varying degrees of SCV–lysosome fusion in macrophages (15,16,38), although these results have been largely ignored, and there are several reasons as to why it may have been missed in epithelial cells. Firstly, compared to professional phagocytes such as macrophages, non-phagocytic cells take up fluid-phase markers inefficiently (39), making it harder to load the amounts required to trace the movement of these markers to and from lysosomes. Secondly, when comparing SCVs with lysosomes, the luminal volume is considerably less, due to the space occupied by *Salmonella*. Therefore, the apparent delivery of fluid-phase markers such as fluorescent dextrans, or lysosomal hydrolases such as cathepsin D, would be expected to be proportionally less. Thirdly, fluid-phase markers, even the fixable dextrans, are largely extracted during processing for immunofluorescence microscopy of fixed cells. Fourthly, much less sensitive detection systems were used in the initial studies of SCV trafficking, which were done over 10 years ago and pre-dated the advent of modern live cell–imaging systems (9). For example, the highly referenced 1995 study by Garcia-del Portillo and Finlay used fixed HeLa cells and was hampered by the limited range of markers available at that time; they used horse radish peroxidase (HRP), revealed by anti-HRP antibodies, as a marker for lysosomal content, and FITC-dextran as a marker for incoming endosomal content. In retrospect, it is easy to point out these limitations but these were seminal studies for the time. In comparison, the spinning disk confocal system used here, in combination with an extremely sensitive charge-coupled device detector and the new generation of fluorescent markers, is ideally suited for extended imaging of living cells, capturing the dynamics of rapidly moving endocytic vesicles and detecting weak fluorescent signals (40). Finally, we cannot exclude the possibility that differences in experimental protocols may contribute to the conflicting conclusions.

The SCV biogenesis model has also been based on the apparent exclusion of lysosomal hydrolases and CI-M6PRs (9,37). Here we have not addressed these issues because we were specifically looking at a live-cell system. However, we have looked at cathepsin D localization in fixed cells and, in agreement with Garcia-del Portillo and Finlay (9), found approximately 30% of SCVs have a clear ‘halo’ of cathepsin D around the bacteria at various time points from 1 to 6 h p.i. (not shown). We think it likely that in unfixed cells, if it were possible to detect endogenous levels of cathepsin D, this number would be significantly higher. Indeed when HK *Salmonella* were internalized into HeLa cells, the number of cathepsin D–positive vacuoles was not significantly different from SCV (not shown). Thus, lack of lysosomal hydrolase reactivity in SCVs may be due more to limitations in the detection, or even low levels of endogenous protein in certain cell types, than active exclusion by *Salmonella*. This does not exclude the possibility that *Salmonella* can modulate interactions with the endocytic pathway. As we have shown here, there does seem to be a delay in acidification of the SCV and an apparent effect on LE/Lys physical interactions. Perhaps this is related to the reported accumulation of Rab7+/LAMP+ vesicles as opposed to cathepsin D+/M6PR+ vesicles, in the vicinity of the SCV (10). But on balance, our data together with previous studies in macrophages (15,16,38) suggest that SCV biogenesis involves sustained interactions with the endocytic pathway including terminal lysosomes.

How does *Salmonella*’s vacuolar biogenesis compare with that of other intracellular pathogens and what does this suggest about the bacterium? For survival in a vacuole, many pathogens avoid the bactericidal action of lysosomes by preventing fusion with these organelles. Some pathogens such as *Chlamydia* spp. do this by segregating completely from the endocytic pathway (41), whereas others including *Legionella pneumophila* and *C. burnetii*, stall the maturation of the vacuole relative to phagosomes containing inert particles or dead bacteria (42,43). But stalling does not mean complete inhibition of fusion, and indeed the *C. burnetii* vacuole fuses promiscuously with endolysosomal vacuoles and ultimately displays many characteristics of a phagolysosome (28,44). Our results suggest that, like *L. pneumophila* and *C. burnetii*, *Salmonella* stalls rather than blocks SCV–lysosome fusion. This delay may allow time for *Salmonella* to adapt to the intracellular environment and establish a foothold for replication by reducing the effectiveness of lysosomal hydrolases delivered to the SCV that require low pH for maturation and activity (45). Evidence in support of this model includes delayed delivery of cathepsin D (37), delayed SCV acidification in epithelial cells (this study) and the finding that dextran-filled lysosomes appear to linger in close proximity to the SCV compared to vacuoles containing *E. coli* (this study). When considering how *Salmonella* might mechanistically delay fusion, type III effectors are the most logical candidates. Indeed, a recent study showed that the T3SS1 effector SopB/SigD modulates SCV biogenesis by altering phosphoinositide metabolism, although, perhaps counterintuitively, SCVs containing mutants lacking SopB showed delayed acquisition of LAMP1 (46). Here we could not detect an overt role for either T3SS1 or T3SS2 in delaying acidification of the vacuole in epithelial cells suggesting that some other *Salmonella* factor is required for this process. Further studies are required to address this important question.

Many intracellular pathogens exploit the host cell microtubule network, and in this respect *Salmonella* is no exception. Microtubules have been shown to mediate three distinct steps in SCV/*Salmonella*-induced filament (Sif) biogenesis: trafficking of the SCV to a juxtanuclear position (47–49), subsequent maintenance of the SCV at a juxtanuclear position (50) and extension of tubular Sifs (51). In addition, reorganization of microtubules in bundles has been observed in close apposition to the SCV (52,53). Here we have shown that microtubules are also required for interactions between the SCV and the endolysosomal system. This may reflect the essential role that microtubules play in endosome/lysosome positioning and movement, but an alternative option is that the retrograde movement of SCVs along microtubules determines its accessibility to endocytic compartments in the perinuclear region (48). Another intriguing possibility is that the microtubule bundles that form adjacent to SCVs (52,53) could somehow be involved in trapping or slowing movement of endocytic vesicles in close apposition to the SCV as we report herein. However, these microtubule bundles have not been detected before 7 h p.i., whereas the fusion events we describe occur within 2–4 h p.i. Ongoing experiments in our laboratory are focusing on later time points and perhaps these will reveal a hitherto undetected connection.

A vital question that arises from this study is, what would be the benefits of SCV–lysosome fusion for *Salmonella*? There are some pathogens, such as *T. cruzi* and *Candida albicans* that actually require lysosome fusion with the parasitophorous vacuole for intracellular survival (54–56). But is this a plausible scenario for *Salmonella*? Certainly, vacuole acidification serves as a signal for SPI2 induction and/or T3SS2-effector translocation and may be necessary for intracellular replication at least in some cell types (34,35,57–61). Another possible benefit of SCV–lysosome fusion would be membrane expansion required to accommodate replicating bacteria and/or the extensive tubular network of Sifs that extend from SCV coincident with the onset of replication (62). It is also likely that the HeLa-cell model used here and in many other studies of *Salmonella*, will not reveal all of the interactions that are important *in vivo*, for example, in polarized intestinal epithelial cells.

In summary, we have shown here that the SCV is accessible to LE/Lys and incoming endocytic traffic by microtubule-, acidification- and Rab7-dependent mechanisms, characteristics of typical endosomal membrane trafficking. Thus, we suggest that the maturing SCV is not an isolated organelle but rather a one that continuously communicates with the endocytic pathway. What truly distinguishes SCV from a phagolysosome remains to be fully answered; however, investigation of the distinguishing trafficking characteristics of different pathogens will continue to shed light on what determines their unique intracellular lifestyles.

## Materials and methods

### Cell culture, bacterial strains and reagents

HeLa (human adenocarcinoma cervix epithelial, CCL-2) and C2BBe1 (human polarized adenocarcinoma colon epithelial cells, CRL-2102) were obtained from the American Type Culture Collection (ATCC, Manassas, VA, USA) and grown in a humidified 37°C, 5% CO_2_ tissue culture incubator. Cell culture reagents were from Invitrogen (Carlsbad, CA, USA) unless otherwise stated. HeLa cells were maintained in Eagle’s Minimal Essential Media (MEM) containing 2 mM l-glutamine, 1 mM sodium pyruvate and 10% heat-inactivated fetal bovine serum (FBS). C2BBe1 cells were maintained in Dulbecco’s modification of MEM (DMEM) containing 4 mM l-glutamine, 0.01 mg/mL human transferrin and 10% FBS. Low passage number (<15 after receipt from ATCC) cells were seeded in six-well dishes and grown o/n before infection.

Wild-type *Salmonella enterica* serovar Typhimurium SL1344 (63) expressing monomeric DsRed (Red-*Salmonella*) under the control of the *Salmonella rpsM* promoter [pFPV-DsRedm/1; (17)] were used for all experiments unless otherwise indicated. The ΔSPI1::kan and ΔSPI2::kan mutants have been described previously (17,64). To create pInv, a plasmid encoding *Yersinia* Invasin, a 4.6 kb fragment encompassing the *inv* locus and approximately 1 kb upstream region was excised from pRI203 (65) by *Bam*HI digestion and ligated into the corresponding site of pWSK129 (66). *Escherichia coli* DH10B (Invitrogen) were electroporated with pFPV-DsRedm/1 (Red-*E. coli*) or both pInv and pFPV-DsRedm/1 (Red-*E. coli* pInv).

All chemicals were from Sigma (St Louis, MO) unless otherwise noted. Stock solutions of bafilomycin A1 (LC Laboratories, Woburn, MA, USA), 200 μM in dimethyl sulfoxide (DMSO) and nocodazole, 20 mM in DMSO, were stored at −20°C. Fluorescent dextrans were from Molecular Probes (Eugene, OR, USA).

### Bacterial growth and infection

To infect epithelial cells, *Salmonella* were grown under conditions to maximize T3SS1-mediated invasion (8). Briefly, o/n shaking 37°C cultures in Luria–Bertani (LB) were diluted in the ratio of 1:33 in fresh LB and grown by shaking at 37°C for 3.5 h to late log phase. *Salmonella* were collected by centrifugation at 5900 × ***g*** for 2 min and resuspended in Hank’s buffered saline solution (HBSS), and used immediately to infect cells (MOI ∼20) for 10 min at 37°C. Extracellular bacteria were then removed by aspiration and monolayers were washed twice in HBSS. Infected cells were incubated for 1 h in growth media (GM) + 100 μg/mL gentamicin to kill extracellular bacteria and then in GM + 10 μg/mL gentamicin for the remainder of the experiment. Heat-killed Red-*Salmonella* were prepared by incubating at 70°C for 10 min. Red-*E. coli* pInv were grown o/n, shaking at 37°C in LB, collected by centrifugation at 5900 × ***g*** for 2 min and resuspended in HBSS. Monolayers were infected with Red-*E. coli* pInv (MOI ∼300) for 30 min at 37°C before washing with HBSS and addition of gentamicin as described above. For co-infection experiments, wt *Salmonella* grown as described above (MOI ∼20) were added for 5 min at 37°C to induce ruffling, the media removed and HK Red-*Salmonella* (MOI ∼14-fold wt) or Red-*E. coli* (MOI ∼17-fold wt) or ΔSPI1 Red-*Salmonella* (MOI ∼7-fold wt) added before centrifuging at 1000 × ***g*** for 5 min at room temperature. Infected monolayers were shifted to 37°C for 5 min before washing with HBSS and addition of GM containing gentamicin as described above.

### Live-cell confocal microscopy

Cells were grown o/n on 25 mm glass coverslips (Fisher Scientific, Pittsburgh, PA, USA) and infected as above. To measure the accessibility of the SCV to lysosomal content, cells were incubated o/n with 230 μg/mL dextran-Alexa Fluor® 488 MW 10 000 or 70 μg/mL dextran-Alexa Fluor® 647 MW 10 000 in GM. Dextran was chased to lysosomes by washing three times in HBSS and incubating cells in dextran-free GM for 3 h prior to infection. Cells were then infected and incubated in CO_2_-independent media containing 10% FBS at 30 min to 8 h p.i. for live-cell imaging. To examine SCV accessibility to incoming endosomal traffic, cells were infected with Red-*Salmonella* before addition of 230 μg/mL dextran-488 or 70 μg/mL dextran-647 at various times p.i. (30 min to 8 h) for 1 h before live-cell imaging. To examine the effect of inhibitors on SCV accessibility, cells were treated as described above except that 1 h before and continuing throughout infection, cells were treated with bafilomycin A1 (0.5 μM) or nocodazole (20 μM) in GM. The SCVs containing fluorescent dextrans were counted and expressed as percentage of SCV accessible to lysosomes or endocytic traffic. All experiments were done at least three times.

For time-lapse live-cell imaging, cells were grown o/n on Delta-T glass bottom dishes (BiopTechs, Butler, PA, USA) before infection with Red-*Salmonella* or Red-*E. coli* pInv as described. Cells were imaged in CO_2_-independent media containing 10% FBS and maintained at 37°C using an objective collar heater and the Delta-T dish controller (BiopTechs). Confocal images were collected using a Yokogawa spinning disk head mounted on a Nikon Eclipse TE2000-S inverted microscope with a 60×, 1.4 NA oil immersion objective (Nikon, Tokyo, Japan). Illumination was provided by a two laser fiber optic laser launcher (Prairie Technologies Inc., Middleton, WI, USA) with krypton/argon (488 nm beam) and argon (568 and 647 nm beams) lasers selected by an acousto-optic tunable filter. Fluorescent emission (filters 525 nm/50, 600 nm/45 and 700 nm/75) was detected using a Photometrics Cascade II:512® camera (Princeton Instruments, Trenton, NJ, USA).

To measure the effect of fixation and immunofluorescence processing on dextran-488 retention in HeLa cells, 200 μg/mL dextran-488 was added o/n to cells grown on 25 mm glass coverslips. Media containing dextran was removed and the cells were washed four times in HBSS and live-cell confocal imaging done in CO_2_-independent media containing 10% FBS. Cells were fixed and processed for immunofluorescence (without antibodies) as described below with the coverslip remaining on the microscope stage in the same position for the entire protocol. Following processing, another image of the same field of view was collected. Quantification of dextran was determined using MetaMorph (Molecular Devices Corporation, Downingtown, PA).

To compare the different dextran internalization procedures, dextran-488 and dextran-568 were internalized into cells via three different protocols. In all cases, HeLa cells grown on glass coverslips were incubated with 400 μg/mL dextran-488 for 4 h and chased o/n in dextran–488 free media to label lysosomes. Dextran-568 (400 μg/mL) was (i) mixed with the dextran-488 and co-internalized for 4 h followed by an o/n chase, (ii) added immediately after the dextran-488 was removed, and incubated o/n and then chased for 3 h in dextran-free media or (iii) added for 20 min immediately before imaging (see Figure S1 for schematic representation). Single optical sections of live cells were collected by confocal microscopy as described below. The percentage of dextran-568 that colocalized with dextran-488 was determined using Volocity (Improvision, Lexington, MA, USA). Briefly, all pixels with a fluorescence value ≥5000 AU (16-bit image) were selected for both images (488 and 568 nm). The percentage of dextran-568 that overlapped with dextran-488 was defined by the sum of all pixels (≥5000 AU) from dextran-568 that also had a value ≥5000 for dextran-488 divided by the sum of all dextran-568 pixels ≥5000, multiplied by 100.

For 3D figures, images were deconvolved using Priism 4.1.2, and reconstructed using Volocity. Time-lapse and 3D movies were made using ImageJ (written by Wayne Rasband at the US National Institutes of Health and available by anonymous FTP from zippy.nimh.nih.gov) and Volocity software, respectively, and all figures assembled using Adobe Photoshop 7.0 (Adobe Systems, San Jose, CA, USA).

### Live-cell vesicle tracking and interaction with the SCV

To examine vesicle association with the SCV and *E. coli* vacuoles, lysosomes were loaded with dextran-488 and infected with Red-*Salmonella* or Red-*E. coli* pInv as described above. Starting at 45 min p.i. images, consisting of three optical sections 0.19 μm apart, were obtained every 3.3 seconds. QuickTime movies were assembled using ImageJ software. To quantify vesicle associations with the vacuole, blind analysis of time-lapse movies examining every fifth frame (21 frames per movie) was done using the Volocity software. Regions of interest (ROI) were selected around the Red-bacteria (*Salmonella* or *E. coli*) and transferred to the corresponding images of dextran-488. Dextran-488 containing vesicles within one pixel (0.19 μm) of each ROI (vacuoles) were counted.

### Measurement of pH_SCV_

HeLa cells grown o/n on 25 mm diameter glass coverslips were incubated with dextran-FITC, MW 10 000 (300 μg/mL) and dextran-647 (75 μg/ml) in normal GM o/n, then infected with Red-*Salmonella* as described, except that fluorescent dextrans were present throughout the infection to maximize delivery to the vacuole. Coverslips were washed in HBSS + 0.5% FBS before live-cell imaging in CO_2_-independent media containing 10% FBS. Confocal images were collected as above. *Salmonella*-containing vacuoles were selected and fluorescence intensity of FITC (488 nm) and Alexa-647 (647 nm) was assessed using MetaMorph software and the ratio 488/647 was calculated (17). To generate a standard curve, the same cells used for experimental measurements were incubated with the ionophore nigericin (20 μM) for 5 min at room temperature, to allow the free exchange of H^+^ ions across membranes, and then with a series of buffers of varying pH (4.0–7.0). The ratio of fluorescence intensity at 488/647 nm was plotted versus buffer pH to produce the standard curve. The pH standard buffers contained 140 mM KCl, 5 mm glucose (15 mM Tris pH 7.0, 15 mM MES pH 6.0 and 15 mM citric acid pH 5.0–4.0).

### HeLa-cell transfections

HeLa cells plated on 25 mm diameter glass coverslips and grown for ∼16 h before transient transfection with the following plasmids using FuGENE6 reagent (Roche, Indianapolis, IN, USA); GFP-Rab7 wt, GFP-Rab7 N125I, GFP-Rab7 Q67L (67), GFP-Rab11 wt, GFP-Rab11 Q70L and GFP-Rab11 S25N (kind gifts from Marino Zerial, Max Planck Institute of Molecular Cell Biology and Genetics, Dresden, Germany). Cells were infected with Red-*Salmonella* 24 h after transfection as described above. After 2 h, GM was replaced with GM containing dextran-647 (120 μg/ml) for 1 h at 37°C. The SCVs containing dextran-647 were counted as described above.

To assess the effect of different chase times on co-localization of internalized dextran with LAMP1, HeLa cells were transfected with LAMP1-mGFP (68) using FUGENE6 reagent. Dextran-568 (400 μg/ml) was internalized via one of two protocols (i) added for 4 h before transfection, and the cells then transfected and incubated o/n in dextran-free media, or (ii) cells were transfected in the presence of dextran with an o/n internalization followed by a 3 h chase. Quantification of the percent dextran-568 that colocalized with LAMP1–mGFP was determined using Volocity as described above except LAMP1–mGFP was used instead of dextran-488.

### Immunofluorescence microscopy

HeLa cells were grown o/n on glass coverslips before transfection and infection as described above. At 1 h p.i., monolayers were fixed in 2.5% paraformaldehyde, pH 7.6, at 37°C for 10 min, followed by extensive washing in PBS. Cells were then permeabilized in PBS containing 10% normal donkey serum (NDS) and 0.1% (w/v) saponin for 10 min at room temperature. Cells were incubated with monoclonal mouse (OSW2) α-116 kDa protein associated with the V-ATPase (Dr Satoshi B. Sato, Department of Biophysics, Faculty of Science, Kyoto University, Kyoto, Japan), in PBS/NDS/saponin for 1 h at room temperature, washed several times in PBS/0.05% (w/v) saponin and then incubated with Cy5-conjugated donkey α-mouse immunoglobulin G (1:1000; Jackson ImmunoResearch Laboratories, West Grove, PA, USA) in PBS/NDS/saponin for a further 1 h. After extensive washing in PBS, coverslips were mounted onto glass slides using Mowiol (Sigma).

### C. burnetii and S. enterica co-infections

HeLa cells were seeded on 12-mm glass coverslips in 24-well plates at 4 × 10^4^ cells/well the day prior to infection. Infection with *Coxiella burnetii* (Nine Mile strain in phase II) clone 4 isolate (RSA 439) was carried out (MOI ∼100) at room temperature for 1 h. The inoculum was then removed, cells were washed and fresh media was added to the infected monolayer. Two days later, *Coxiella*-infected HeLa cells were infected with T3SS1-induced *Salmonella* as described above (MOI ∼50). At 2 h and 6 h p.i., monolayers were fixed in 2.5% paraformaldehyde, permeabilized and immunostained with the following antibodies: guinea-pig α-*Coxiella* [(69), 1:1000], rabbit α-*Salmonella* lipopolysaccharide (1:1000, Difco, Franklin Lakes, NJ), mouse α-LAMP1 (H4A3, 1:1500; Developmental Studies Hybridoma Bank, Iowa City, IA, USA). Alexa Fluor® 488- and 568-conjugated antibodies (Molecular Probes) and cyanine5-conjugated antibodies (Jackson ImmunoResearch) were used at a final dilution of 1:800. Coverslips were mounted onto glass slides with Mowiol and samples were viewed by fluorescence microscopy. The number of co-infected cells with *Salmonella* and *Coxiella* in the same vacuole was scored. Results are the mean ± SD from three independent experiments with ≥100 co-infected cells scored in each experiment. Images are Z-projections of 12–15 optical sections.

## Supplementary Materials

Figure S1 Optimization of conditions for labelling and imaging LE/Lys. A) Loss of fluid-phase dextran-488 after fixation and processing for immunofluorescence microscopy. The panel on the left shows the endocytic pathway labeled with dextran-488 in live cells. The panel on the right shows the same field of view after fixation and processing (see Materials and Methods). Below each panel is a graph of pixel intensity along the red line in the micrographs (arrows at either end) illustrating the loss of signal after processing. B) To compare internalization protocols for labeling LE/Lys dextran-488 and dextran-568 were internalized into HeLa cells. In all three protocols, dextran-488 was internalized for 4 h and then chased to lysosomes by o/n chase. Dextran-568 was (i) co-internalized with dextran-488 for 4 h (to give maximal co-localization), (ii) added immediately after dextran-488 was removed, and internalized o/n then chased for 3 h in dextran-free media or (iii) added for 20min immediately before imaging (see Figure S1 for schematic representation). The percent dextran-568 that colocalized with dextran–488 containing lysosomes was then determined from images acquired during live-cell imaging. In separate experiments, cells were transfected with LAMP1-mGFP and then dextran-568 was internalized for either (i) 4 h and chased o/n in dextran-free media, or (ii) o/n and chased in dextran-free media for 3 h before imaging. All values are the mean ± SD from one representative experiment.

Video S2 Three-dimensional image volume rendering of SCVs containing Red-Salmonella (blue) showing content mixing of dextran-647 (red) delivered from lysosomes and dextran-488 (green) from incoming endocytic traffic. HeLa cells, 3 h p.i. See Figure 2B for details.

Video S3 Time-lapse series showing accumulation of the lysosomal content marker dextran-488 (green) in a single SCV containing Red- Salmonella (red). Confocal live-cell imaging was initiated at 18 min p.i.(00:00) acquiring single optical frames every 3 seconds. Play back speed 6 frames per second. See Figure 2D for details.

Video S4 Time-lapse series showing interactions of dextran-488 loaded lysosomes (green) with SCVs containing Red-Salmonella (red) in HeLa cells. Confocal live-cell imaging was initiated at 45 min p.i. acquiring single optical frames every 0.7 seconds. See Figure 5 for details.

Video S5 Time-lapse series showing interactions of dextran-488 loaded lysosomes (green) with vacuoles containing Red-E. coli pInv (red) in HeLa cells. Confocal live-cell imaging was initiated at 45 min p.i. acquiring single optical frames every 0.7 seconds. See Figure 5 for details.

Video S6 Three-dimensional image volume rendering of Salmonella (red) at 6 h p.i. residing in a C. burnetii (green) parasitophorous vacuole stained for LAMP1 (blue) in co-infected HeLa cells. See Figure 6 for details.

Supplemental materials available as part of the online article from http://www.blackwell-synergy.com

Salmonella trafficking is Defined by Continuous Dynamic Interactions with the Endolysosomal System

D Drecktrah, LA Knodler, D Howe and O Steele-Mortimer

The supplemental materials for this materials is available through the Traffic home page at: http://www.traffic.dk/suppmat/8_3e.asp

These supplemental data are provided by the authors, and hosted on the Traffic home page. Blackwell-Munksgaard maintains and operates this site.
