# Supplementary figures and images for: Salmonella Trafficking is Defined by Continuous Dynamic Interactions with the Endolysosomal System
Source: Traffic. 2007 Jan 15;8(3):212–25. doi: 10.1111/j.1600-0854.2006.00529.x (PMC2063589; doi:10.1111/j.1600-0854.2006.00529.x)

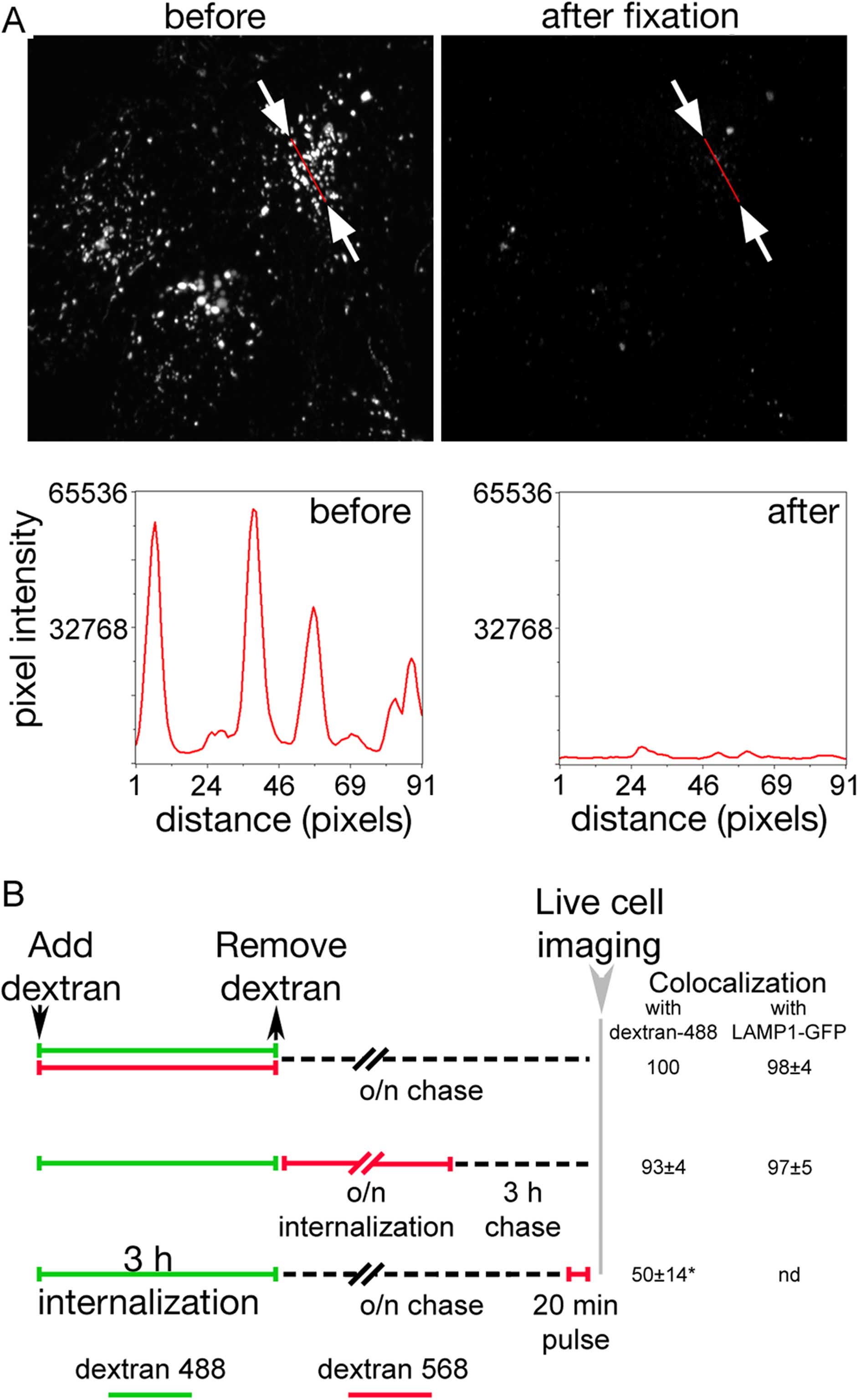

Supplement: Figure S1 — Optimization of conditions for labelling and imaging LE/Lys. A) Loss of fluid-phase dextran-488 after fixation and processing for immunofluorescence microscopy. The panel on the left shows the endocytic pathway labeled with dextran-488 in live cells. The panel on the right shows the same field of view after fixation and processing (see Materials and Methods). Below each panel is a graph of pixel intensity along the red line in the micrographs (arrows at either end) illustrating the loss of signal after processing. B) To compare internalization protocols for labeling LE/Lys dextran-488 and dextran-568 were internalized into HeLa cells. In all three protocols, dextran-488 was internalized for 4 h and then chased to lysosomes by o/n chase. Dextran-568 was (i) co-internalized with dextran-488 for 4 h (to give maximal co-localization), (ii) added immediately after dextran-488 was removed, and internalized o/n then chased for 3 h in dextran-free media or (iii) added for 20min immediately before imaging (see Figure S1 for schematic representation). The percent dextran-568 that colocalized with dextran–488 containing lysosomes was then determined from images acquired during live-cell imaging. In separate experiments, cells were transfected with LAMP1-mGFP and then dextran-568 was internalized for either (i) 4 h and chased o/n in dextran-free media, or (ii) o/n and chased in dextran-free media for 3 h before imaging. All values are the mean ± SD from one representative experiment. [file tra0008-0212_fig_s1.jpg]
